# Sleep memory processing: the sequential hypothesis

**DOI:** 10.3389/fnsys.2014.00219

**Published:** 2014-12-16

**Authors:** Antonio Giuditta

**Affiliations:** Department of Biology, Federico II UniversityNaples, Italy

**Keywords:** synaptic homeostasis, slow wave sleep, REM sleep, memory processing, memory storage, quantum hypothesis

## Abstract

According to the sequential hypothesis (SH) memories acquired during wakefulness are processed during sleep in two serial steps respectively occurring during slow wave sleep (SWS) and rapid eye movement (REM) sleep. During SWS memories to be retained are distinguished from irrelevant or competing traces that undergo downgrading or elimination. Processed memories are stored again during REM sleep which integrates them with preexisting memories. The hypothesis received support from a wealth of EEG, behavioral, and biochemical analyses of trained rats. Further evidence was provided by independent studies of human subjects. SH basic premises, data, and interpretations have been compared with corresponding viewpoints of the synaptic homeostatic hypothesis (SHY). Their similarities and differences are presented and discussed within the framework of sleep processing operations. SHY’s emphasis on synaptic renormalization during SWS is acknowledged to underline a key sleep effect, but this cannot marginalize sleep’s main role in selecting memories to be retained from downgrading traces, and in their integration with preexisting memories. In addition, SHY’s synaptic renormalization raises an unsolved dilemma that clashes with the accepted memory storage mechanism exclusively based on modifications of synaptic strength. This difficulty may be bypassed by the assumption that SWS-processed memories are stored again by REM sleep in brain subnuclear quantum particles. Storing of memories in quantum particles may also occur in other vigilance states. Hints are provided on ways to subject the quantum hypothesis to experimental tests.

## Introduction

The recent publication of an extended review article on sleep memory processing (the synaptic homeostatic hypothesis or SHY; Tononi and Cirelli, 2014) has suggested a comparison of its premises, data, and overall perspective with the corresponding viewpoints of the sequential hypothesis (SH) that was proposed more than 30 years ago (Giuditta, 1977, 1985) but is now largely marginalized. SH was the first to examine and demonstrate the involvement of slow wave sleep (SWS) in memory processing, and to point out its primary role in downscaling irrelevant or competing memory traces. The two-step mechanism of memory processing proposed by SH was concluded by the renewed storage of SWS-processed memories during REM sleep (Giuditta et al., 1995, 2003; Ambrosini and Giuditta, 2001). These views were originally demonstrated in rats, and later confirmed by independent investigations of human subjects (Ficca et al., 2000; Gais et al., 2000; Stickgold et al., 2000).

Comparing SHY with SH has not been attempted so far despite their similar premises and their different interpretations of the role of SWS and REM sleep. Similarities obviously include the involvement of SWS in memory processing, and in its weakening or elimination of memory traces. The latter operations were later raised by SHY to the rank of a primary homeostatic function assumed to fulfill the main goal of sleep. Most differences appear to have roots in different views of brain activity that is largely random for SHY but well ordered for SH.

The current predominance of SHY in interpreting the role of sleep has not prevented a good deal of criticism mainly stressing SWS capacity to potentiate synaptic strength and consolidate memories (Diekelmann and Born, 2010; Timofeev, 2011; Frank, 2012, 2013, 2014; Ribeiro, 2012; Abel et al., 2013).

## A brief summary of SHY

According to SHY, environmental regularities captured by brain during waking strengthen synaptic connections, increase plastic supplies and energy needs, and saturate learning capacity. Conversely, in the ensuing sleep most changes are reversed, and acquired memories are integrated within the vast repertoire of previous memories. Support for a net synaptic potentiation during waking and a net synaptic depression during sleep was provided by a wealth of biochemical, morphological and neurophysiological data (Tononi and Cirelli, 2003, 2006, 2014). Sleep effects were attributed to delta EEG waves in view of their elevated intensity at sleep onset, and of their progressive increase after prolonged waking. Further support came from their decrease with time asleep and after naps, and from their specific increase in defined brain regions involved in selected learned tasks. According to SHY the process of synaptic renormalization during sleep fulfills the homeostatic need to reset experience-modified brain synapses to their native state. This process renews brain capacity to acquire information in the following waking period. In short, “sleep is the price the brain pays for plasticity”.

## The genesis of SH

At the time the SH was envisaged (Giuditta, 1977, 1985) sleep literature was redundant with hypotheses regarding the role of REM sleep (synonymous with paradoxical sleep or PS) and SWS (synonymous with NREM or non rapid eye movement sleep). They were considered functional states serving widely different roles. Most attention was paid to REM sleep in view of its involvement in learning (Fishbein and Gutwein, 1977; Pearlman, 1979) but also because of its elevated brain metabolism and unique relation with dreams. Completely different roles were assigned to SWS. They included restoration, anabolism, energy saving, etc. (Allison and Cicchetti, 1976; Adams and Oswald, 1977; Horne, 1977; Walker and Berger, 1980; Shapiro et al., 1981).

Despite their presumed divergent roles, REM sleep and SWS were not independent sleep stages. SWS started the sleep period of the adult mammal long before REM sleep, and markedly prevailed in duration; it also displayed sharply different features from REM sleep and from waking. In addition, waking experience modulated either type of sleep: REM sleep by memory acquisition, SWS by the load of visual percepts (Horne and Walmsley, 1976), the intensity of physical exercise (Horne, 1981), or the novelty and stress of experience (Reich et al., 1972). The latter observation was of relevance for the genesis of the SH since it demonstrated that brain energy needs during SWS depended on the nature of the previous waking period. Indeed, the concentrations of brain lactate and pyruvate remained essentially unchanged during quiet or active waking but markedly differed in the following SWS. They slightly decreased after quiet waking but markedly increased after active waking. The data suggested that active waking induced a special brain condition that could only be renormalized at energy expenses during the following SWS.

Additional considerations regarded the ontogenetic development of sleep. In mammals sleep appears in late fetal life as a primitive form of REM sleep (active sleep) that persists in the newborn while progressively assuming adult features. On the other hand, waking and SWS make their appearance in the newborn and start differentiating into mature states. Notably SWS periods that initially occur at random, start positioning themselves between waking and REM sleep. These modifications suggested that in early ontogenetic stages active sleep promoted the laying down of basic brain circuits that were elicited by innate instructions (Roffwarg et al., 1966) while in later developmental stages brain growth started to be molded by waking experience. This drastic switch in the source of information implied that, at variance with innate instructions, waking experience also contained irrelevant or inappropriate information that was to be cleared to prevent interference with the further growth of brain circuits. In view of its strategic position between waking and REM sleep, SWS appeared to be well fit to perform this cleaning operation before the final memory storage promoted by REM sleep (Giuditta et al., 1995). This scenario was in agreement with the logic of food acquisition also requiring a differential processing of what is to be retained from what is to be eliminated. Brain appeared to adopt the same formal logic with regard to newly acquired information.

These considerations led to the proposal that memory processing did not only involve REM sleep, as generally believed, but also included SWS to preliminarily sort memories to be retained and weaken or eliminate remaining traces. Accordingly, waking memories were assumed to be initially trimmed of irrelevant, non adaptive, or competing traces during SWS, and to be eventually stored again in better format during REM sleep. The latter step was also promoting the integration of retained memories with preexisting memories (Giuditta, 1977, 1985; for reviews, see Giuditta et al., 1995, 2003; Ambrosini and Giuditta, 2001). In this new perspective, the operations performed by SWS were assumed to take memories into a state comparable to that of innate instructions. Overall, the flow of information from waking to REM sleep was summarized as I_W_->I_SWS_->I_REM sleep_.

## Testing SH

The validity of SH was tested in a variety of training conditions that mostly concerned adult rats exposed to a two way active avoidance task (Giuditta et al., 1985; Ambrosini et al., 1988, 1992, 1995; Langella et al., 1992). In the initial experiments (Giuditta et al., 1985) EEG analyses demonstrated that post-trial SWS episodes were markedly longer in either learning and non learning (NL) rats with respect to baseline sleep and to control rats. However, lengthening of SWS episodes markedly differed in the two groups depending on their following episode that could be waking (SWS->W) or REM sleep (SWS->REM sleep). In learning rats SWS episodes initially lengthened during SWS->W sequences and much later in SWS->REM sleep sequences. Conversely, in NL rats the same effect mostly concerned SWS->W sequences. Furthermore, the number of REM sleep episodes only increased in learning rats. As predicted by SH, the participation of SWS in memory processing started much earlier than that of REM sleep. The data demonstrated that REM sleep was not the only sleep involved in memory processing. Rather, SWS episodes increased their duration while REM sleep episodes increased their number. Differences between learning and NL rats appeared to reflect the processing of avoidance memories that prevailed in the former group, and the processing of innate memories that prevailed in the latter group. Interestingly, comparable differences between learning and NL rats were also detected in baseline sleep (Ambrosini et al., 1993). This suggested that to a certain extent rats were innately conditioned to learn the avoidance task.

In these early studies the experimental protocol included a training day and a previous baseline day during which sleep was EEG recorded at the same time (about 1.00 pm) and for the same duration (3 h) of post-trial sleep. The training session lasted 4 h and included 4 training periods of 30 min separated by resting periods of the same duration. Rats were assigned to a fast learning (FL) or to a NL group depending on their attaining the learning criterion. In later experiments (Ambrosini et al., 1988, 1992, 1995; Langella et al., 1992) a test session was added on the third experimental day to assess the long-term retention of the task. The addition revealed that the NL group also included rats that attained criterion the third day (slow learning or SL rats) together with rats that persisted in their failure to learn (NL* rats). SL rats belonged to the category of reminiscent animals which attain the learning criterion after a long delay but without additional training (Anderson, 1940; Huppert and Deutsch, 1969; Jaffard et al., 1974). In our hands, lengthening of SWS episodes in SL rats only occurred in the third hour of post-trial sleep and the number of REM sleep episodes only increased in the sixth hour (Ambrosini et al., 1995).

In these later studies EEG records were analyzed with a higher time resolution (1 s) that allowed the identification of brief episodes of transition sleep (TS) that were previously missed. In a TS episode the delta waves of a previous SWS episodes suddenly mix with theta and alpha waves. Their identification led to the definition of two additional sleep sequences (SWS->TS->W and SWS->TS->REM sleep) and to the more accurate identification of SWS->W and SWS->REM sleep sequences previously lumped together with related TS-containing sequences. These improved analyses revealed that in the post-trial sleep of learning rats longer SWS episodes initially occurred in SWS->TS->W sequences and eventually in SWS->TS->REM sleep sequences. Conversely, in NL rats longer SWS episodes largely occurred in SWS->W and SWS->REM sleep sequences. Hence, avoidance memories appeared to be processed during TS-containing sequences, while innate memories were processed during sleep sequences lacking TS. Comparable differences were present in baseline sleep of FL, SL and NL* rats (Vescia et al., 1996; Mandile et al., 2000). This further indicated that rapidly learning, slowly learning, and NL rats were partly conditioned by their respective innate tendencies.

The identification of different sleep sequences that started with an apparently similar SWS episode but were selectively involved in processing adaptive or innate memories suggested that the SWS episodes were differing from each other, and that their differences conditioned the appearance of the following sleep episode (TS, W, or REM sleep). Comparable differences were presumed to regard TS episodes. In addition, SWS differences appeared to be generated by the behavioral responses prevailing in FL, SL, and NL* rats, in full agreement with SH premises and with the constraints of an ordered brain activity. In view of the basic differences in nucleic acid metabolism exhibited by waking and sleeping brains (see below), SWS differences seemed related to the nature of newly synthesized nucleic acids.

Differences in sleep episodes and sleep sequences were eventually extended to the large clusters of baseline sleep sequences (trains) that were bordered by relatively long (>60 s) W episodes (Piscopo et al., 2001). Analyses of such baseline structures allowed the identification of mixed and homogeneous trains. The former trains contained all the four sleep sequences and was most abundant; they were labeled +TSW trains in view of the SWS->TS->W sequence they contained that was lacking in the less abundant −TSW trains. Homogeneous trains were least abundant. Baseline trains were not artificial entities lacking meaningful roles since they were non randomly distributed in FL, SL and NL* rats. Variables of +TSW trains and of their sleep sequences and episodes were prevalent in FL rats; they also exhibited significant correlations with the avoidances scored the following day. Conversely, variables of −TSW trains and of their sleep sequences and episodes were prevalent in NL* rats; they correlated with freezings rather than with avoidances (Piscopo et al., 2001). Interestingly, the role of sleep sequences depended on the train that included them. This was best shown by variables of SWS->TS->REM sleep sequences that correlated with avoidances if present in +TSW trains, but correlated with freezings (and inversely correlated with avoidances) if present in −TSW trains. Variables of homogeneous trains also exhibited specific correlations with behavioral responses. Those of homogeneous SWS->TS->W trains directly correlated with avoidances while those of homogeneous SWS->W trains inversely correlated with avoidances.

An additional observation that is worth reporting concerned the increment in delta waves that was selectively present in FL rats while they were performing in the initial training period of the two way active avoidance task. This unexpected effect significantly contributed to their selectively attaining the learning criterion in that period (Mandile et al., 2003). It was also observed that the SWS->TS->W sequence that consistently initiates processing of avoidance memories by displaying longer SWS episodes, was absent in the post-trial sleep of FL rats. Surprisingly the SWS->TS->REM sleep sequence that normally concludes avoidance processing (Mandile et al., 2000) was instead present. It appeared that the lack of the former sequence in post-trial sleep was due to the selective increment in delta waves present in performing FL rats that presumably fulfilled the same role.

## Brain nucleic acid metabolism in waking and sleep

In adult rabbits monitored by EEG recording newly synthesized brain cortex RNA contained a larger proportion of ribosomal RNA if rabbits were prevalently awake, but a larger proportion of heterogeneous nuclear RNA if they were mostly sleeping (Vitale-Neugebauer et al., 1970). Comparable results were obtained with purified large nuclei from neurons and astrocytes that also exhibited a marked increment in the content of newly synthesized RNA when rabbits were mostly asleep. These effects were absent in purified small nuclei presumably derived from oligodendrocytes and small neurons. Increments in newly synthesized RNA also occurred in nuclear fractions from purified neuronal perikarya and partially purified glial cells (Giuditta et al., 1980).

Direct evidence that brain nucleic acid metabolism is modulated by post-trial sleep was provided by comparing rat brain DNA synthesized during a training session before and after post-trial sleep. At the end of the training session the content of newly synthesized brain DNA was about the same in learning rats scoring a high number of avoidances and in NL rats receiving a high number of foot-shocks triggered by their innate responses (Giuditta et al., 1995). On the other hand, major differences were present after post-trial sleep since half of the newly synthesized DNA was selectively lacking in NL rats displaying an increment in REM sleep episodes with regard to baseline sleep (Giuditta et al., 1985; Langella et al., 1992). In the same rats newly synthesized brain DNA inversely correlated with variables of post-trial SWS->REM sleep sequences. These effects were absent in learning rats. Data were interpreted to indicate that brain DNA synthesized during the training session was a molecular correlate of acquired memories. As such, its recovery after post-trial sleep depended on the fate of those memories: the DNA associated with the avoidance memories prevailing in learning rats was retained while that associated with the innate memories prevailing in NL rats was largely eliminated. These processes occurred during the post-trial SWS->REM sleep sequences of NL rats as shown by their inverse correlation with newly synthesized brain DNA.

These experiments were suggested by data demonstrating the high turnover of brain DNA in adult rats (Perrone Capano et al., 1982) and its modulation by a variety of activities (Pelc and Viola-Magni, 1969; Pelc, 1972) and learning protocols (Reinis and Lamble, 1972; Ashapkin et al., 1983; Giuditta et al., 1986). Brain metabolic DNA is distributed in brain cellular and subcellular fractions and is highly dispersed throughout the genome in a learning-dependent pattern (Giuditta et al., 1986). These features exclude its involvement in neurogenesis, gliogenesis, or DNA repair. Rather, they strongly suggest its participation to the acquisition and processing of novel information.

## Comparing SHY with SH

As briefly mentioned in the Introduction, SHY and SH share some basic similarities but markedly differ in experimental methods and results, and in their interpretation and significance. Differences between the two hypotheses are numerous and basic as they touch the essence of brain activity and the role of sleep in memory processing. Accordingly, they are being presented and discussed one at a time in the following list of topics.

### Brain energy needs

A first comment regards SHY’s assumption that brain energy needs are high in waking due to synaptic potentiation, and low in SWS due to synaptic depression. The former process may well require some extra energy but it does not follow that synaptic depression may need less. Rather, the concentration of brain energy metabolites indicates that an opposite situation may prevail (Reich et al., 1972). Indeed, the content of brain lactate and pyruvate in active waking did not differ from quiet waking, but markedly increased in the ensuing SWS. No such increase occurred in SWS after a period of quiet waking. Since synaptic potentiation is likely to prevail in active waking, these observations are at variance with SHY’s expectation. It should also be noted that the above data are in agreement with the values of total EEG spectral power (µV^2^) determined in adult rats. They are almost the same in active and quiet waking but attain markedly higher values during SWS (Ambrosini et al., 1994).

### The role of sleep in memory processing

SH was concerned with the differential processing of memories during sleep, and highlighted the adaptive role of retained memories. On the other hand SHY focused interest on the homeostatic significance of sleep synaptic renormalization. Attention was largely devoted to the necessary turnover of synaptic strength and to its significance in re-establishing the conditions for memory acquisition in the following waking period. This goal is of obvious relevance for the wellbeing and survival of organisms, but the grand objective of sleep remains the retention of selected memories and their integration with preexisting memories. The homeostatic role of synaptic renormalization should not be minimized but at the same time its emphasized significance should not marginalize the differential processing of memories occurring during SWS and their diverging paths of retention or elimination. These considerations make SHY fall short of being the ultimate hypothesis on sleep memory processing.

An implicit more basic difference between SHY and SH regards brain activity. Its nature is largely random by SHY but deeply ordered for SH. Accordingly, the net modifications of synaptic strength reported by SHY in waking and sleep are the result of a random approach that leaves in the background the significance of retained memories. Synaptic renormalization is also touching a key problem that seems to have escaped notice. If modified synapses are renormalized during sleep, what happens to the memories they store? This question will be discussed in more detail further on. For the time being let it be emphasized that sleep memory processing is likely to include the following operations: (i) tagging memories to be retained; they are often adaptive memories but may also be innate memories; (ii) retaining the tagged memories and concurrently weakening or eliminating the remaining memory traces; (iii) integrating the retained memories within the much larger population of preexisting memories. These operations are likely to elicit a quicker and more specific retrieval of memories during waking, and possibly from a wider repertoire of percepts. Sleep memory processing might also play a role in transferring compacted memories to germ cells as epigenetic modifications (Rudenko and Tsai, 2014) or according to a Lamarckian mechanism of brain evolution (Barry, 2013).

### SHY’s experimental approaches

Memories acquired during waking are unlikely to be exclusively stored in clusters of potentiated synapses. Neural circuits are frequently modified by waking experiences that down modulate synaptic strength. It follows that synaptic renormalization during sleep cannot only downscale potentiated synapses but is likely to also include reinforcing weak synapses. This consideration brings to light a major limit of the random methods used to assess the net strength of heterogeneous synaptic populations (Tononi and Cirelli, 2014). They provide statistical information unable to reveal the origin of the assessed changes, whether they derive from potentiated or normal synapses, or from adaptive, innate, or irrelevant memories. It may also be worth noting that after a waking experience brain activity keeps on acquiring new percepts in addition to processing waking percepts. Notably during sleep brain cortical regions are not exclusively involved in processing environmental information; they are also busy receiving and processing stimuli largely derived from the gastrointestinal tract (Pigarev, 1994; Pigarev et al., 2013; Pigarev and Pigarev, 2014). It follows that heterogeneous synaptic populations analyzed by random methods may include synaptic elements that are not related to waking memories.

SHY’s preference in attributing net changes in synaptic strength to waking and sleep rather than to memories acquired during waking or processed during sleep appears indulging in the view that brain activity is largely random. This may justify the random methods used and the claim that “sleep is the price paid to plasticity”, but cannot account for the selective changes in synaptic strength supporting memory acquisition and the selective modifications they undergo during sleep. There are reasons to believe that the role of sleep in memory processing may be worth a more generous evaluation.

At variance with SHY, the modifications undergone by adaptive or innate memories during sleep may be followed with the procedures adopted by SH. In addition to high resolution EEG analyses they include behavioral methods and the monitoring of brain macromolecules synthesized during training before and after post-trial sleep (Giuditta et al., 1985, 1995; Langella et al., 1992). This protocol allows the correlative analysis of memories with variables of post-trial sleep, and with brain macromolecules synthesized during the training session. More incisive information may be provided by investigating the selective pattern of genomic expression prevailing in sleep (Mackiewicz et al., 2007), or by monitoring the newly synthesized proteins selectively triggered by learning in the local synaptic system of gene expression (Eyman et al., 2013) or the activated synaptic RNAs (Ferrara et al., 2009). The local system of synaptic gene expression is supported and modulated by the intercellular transfer of perisynaptic glial transcripts to nerve terminals (Giuditta et al., 2008). These data might shed some light on the macromolecules that are presumably conditioning post-trial SWS episodes in their participation to sleep sequences selectively involved in processing novel or innate memories.

### Integration of SWS-processed memories

An additional key difference between SH and SHY regards the mechanism of integration of retained memories in the larger population of preexisting memories. According to SH, memories processed during SWS are stored again as core memories during REM sleep that concurrently integrates them in the wider realm of preexisting memories (Giuditta et al., 1995, 2003; Ambrosini and Giuditta, 2001). This additional storage step was attributed to REM sleep in view of the similar but not identical features it shares with waking which is the brain state in which memories are initially acquired and are most frequently recalled. Their common features might also facilitate memory retrieval during waking. These considerations are largely ignored by SHY that attributes the integration of new memories with preexisting memories to the comprehensive sampling of statistical regularities in brain activity occurring during SWS. What role is left for REM sleep? In their recent review (Tononi and Cirelli, 2014) REM sleep is even doubted to have a role in memory processing, presumably in view of the pervading capacities attributed to SWS. This peculiar stand is turning upside down in a somewhat ironical way the initial considerations that made REM sleep the first and only sleep involved in memory processing (Fishbein and Gutwein, 1977; Pearlman, 1979).

### Memory storage and the role of REM sleep

According to SHY, brain synapses potentiated during waking undergo renormalization in the ensuing SWS to prevent the experience-dependent impoverishment of native synapses that is needed to implement learning capacity. Since SHY’s random methods cannot identify which memories are supported by renormalized synapses, it may be conveniently assumed that they do not belong to the category of retained memories. If they did, those memories would be lost. Since this ill-omened consequence was rarely reported, the consequent worrying scenario may be discounted. Nonetheless, a comparable scenario might be in sight even if renormalized synapses consistently supported memories not worth retaining. Indeed, given the widely held assumption that long term storage of memories may only be implemented by modifications of synaptic strength, modified synapses supporting retained memories would accumulate in brain with each waking/sleep cycle. Consequently the number of native synapses available for learning would be reduced.

Synaptic potentiation and depression cannot be doubted, but their persistence in time remains largely undefined. Even assuming their lifelong existence, would the number of native synapses be sufficient for storing all available memories? Answers to this question are likely to be based on shaky premises and may only lead to uncertain outcomes. What may not be debatable is the expectation that given the present SHY formulation and the accepted mechanism of memory storage, modified synapses would start accumulating in brain since an early age, thereby interfering with learning capacity. The timing of such outcome may be questionable, but the end result could not. The problem may also be examined from a different point of view. When memories of past events are retrieved after long time intervals (months, years, or decades), does this imply that the same (or an equivalent) configuration of modified synapses persisted? How strong is the evidence that modified synapses might survive such long times? Are we sure that other storing mechanisms do not exist?

If properly considered, the latter question might prompt a search for an additional mechanism possibly endowed with a much larger capacity for information storage. Before attempting to deal with such a problem it may be of value to compare SHY with other homeostatic systems governing organisms. Their general goals are similar. They have to counteract changes in biological set values as soon as triggering conditions are sensed. This mechanism is serving a positive role in a multitude of biological processes but might not be blindly adopted when homeostatic variables regard basic features of learning capacity as brain modifiable synapses. In the latter case re-establishing homeostatic set values under the currently accepted framework of memory storage is likely to interfere with the same learning capacity needing protection. SHY did not ignore this constraint but failed to take it to its extreme consequences. The two horns of the dilemma were considered but adopted responses did not lead to a suitable solution. Memories were partly saved, synapses were partly renormalized but this accommodating attitude failed to consider that retained memories would progressively accumulate with each sleep/waking cycle, and thereby deprive the reservoir of native synapses. Memories are known to persist for the entire life of an organism (Mariucci et al., 1998), and in some human subjects they may attain an incredibly large load (LePort et al., 2012). Would modified synapses persist for such a long time and to such a large extent without hindering learning capacity?

Not so long ago the philogenetic increase in brain size was attributed to the accumulation of memories (Crick and Mitchison, 1983, 1995), and their storage mechanism was discussed in the perspective of a generalized molecular turnover (Crick, 1984). Brain size was assumed to be kept under control by the elimination of “parasitic” memories during REM sleep in a process of “reverse learning”. These speculations call attention to the striking philogenetic increase in brain size. Was it really due to an increment in the load of memories? Could this suggest that memories might be transferred to the progeny? Epigenetic memories are known to cross the trans-generational barrier (Rudenko and Tsai, 2014) but no specific reference was made to brain size. What could be a satisfying solution?

### The quantum hypothesis

In principle an additional site of memory storage should fulfill the twin requirement of being located near brain circuits while occupying a much smaller fraction of their allotted space. An attractive option is offered by the vanishingly small size and peculiar quantum properties of subnuclear brain particles. Quantum computers feature an unbelievably huge storage capacity and an incredibly fast computation rate. Quantum physicist Richard Feynman is known to say: “there’s plenty of room at the bottom”. This intriguing option is however running against the generalized opinion that biological features derive from macromolecular components that are often considered the ultimate source of biological capacities. This view is clearly lacking logical support. No compelling reason was ever proposed that prevented submolecular components from contributing to biological capacities (Giuditta, 2012). Indeed, quantum features are increasingly considered as potential intermediates in a number of biological processes that include the photosynthetic reaction and even consciousness (Dawlaty et al., 2012; for a review, see Hamenoff and Penrose, 2014). Why should subnuclear particles be unable to store brain memories? Information might quickly reach them from modified synapses, and likewise be quickly retrieved in a kind of seesaw between different levels of the same entities. Notably, subnuclear particles are the ultimate components of brain synapses as they are of everything else in the universe (Giuditta, 2012).

The quantum hypothesis is clearly lacking a mechanism, but mechanisms start to be investigated only after hypotheses reach experimental consistency. SHY’s unsolved dilemma requires that the present mechanism of memory storage be supplemented by an additional high capacity mechanism allowing full synaptic renormalization without memory loss. The quantum hypothesis offers a possible alternative that is worth considering. One may then ask at what stage of sleep information might be transferred to subnuclear particles. Most likely such step would not occur before SWS processing is completed. Hence, memory transfer in mammals and birds might take place during REM sleep. The proposal is in agreement with SH view of REM sleep concluding the two-step memory processing by promoting the renewed storage of SWS-processed memories and integrating them with preexisting memories (Giuditta et al., 2003). In the framework of the quantum hypothesis, REM sleep would still promote the storage of SWS-processed memories but in brain subnuclear particles rather than in modified neural circuits. Interestingly, this novel location would assume additional relevance by the plausible suggestion that preexisting memories may also been stored in comparable locations, in line with the hypothesis of a phylogenetic origin of human mind (Giuditta, 2012).

The role of REM sleep in the adult organism should also be compared to that of its precursor active sleep, and to its proposed involvement in the laying down of basic brain circuits (Roffwarg et al., 1966; Giuditta et al., 1995). The latter operations are assumed to be guided by innate instructions despite the substantial lack of knowledge regarding their genomic or epigenomic nature, and the transferring modalities to the growing brain. In view of the intimate relation between active sleep and REM sleep, one may wonder whether innate instructions are somehow related with the new knowledge memorized by parents when learning to grow new brain circuits. Some kind of connection might only be envisaged in the framework of a Lamarckian mechanism of brain evolution (Barry, 2013). While such questions are likely to remain without suitable answers for some time, it may still be worth noting that deprivation of active sleep in fetal rats induces a marked increment in brain DNA synthesis (Grassi Zucconi et al., 1986).

### Testing the quantum hypothesis

The hypothesis of a REM sleep-mediated transfer of memories from neural circuits to brain subnuclear particles might be tested in relatively simple but less direct analyses, and in more complex but direct experiments. In the former approach, rats or mice trained for a complex task should be selectively deprived of REM sleep in their post-trial period. In comparison with trained animals allowed normal sleep they may be expected to exhibit a higher net degree of synaptic potentiation presumably due to an impaired memory transfer to subnuclear particles. Alternative interpretations should be examined by training animals for different tasks, including those involving the participation of local brain regions. These experiments should also be made as a follow up response to the observation that “many of the findings suggestive of renormalization were obtained in relation to total sleep, not just NREM sleep” (Tononi and Cirelli, 2014). The possibility that REM sleep might contribute to the renormalization process finds support in the marked loss of newly synthesized brain DNA during the post-trial sleep of NL rats exhibiting an increment in REM sleep episodes (Giuditta et al., 1985). Notably, the content of newly synthesized brain DNA inversely correlated with variables of SWS->REM sleep sequences, and was attributed to a related loss of memories (Langella et al., 1992). Investigations on the participation of REM sleep in synaptic renormalization are still lacking. They might reveal its specific involvement in the homeostatic process but would not demonstrate memory transfer to subnuclear brain particles.

In more direct tests trained animals should be exposed to the intense magnetic field of fNMR machines during post-trial sleep or, more selectively, during REM sleep. Since these fields interfere with the spin of subnuclear brain particles, this specific alteration might prevent memory transfer. Trained animals should also be exposed to the magnetic field during SWS or waking, and to the fNMR machine when magnetic field is not in operation. Interference with the spin of brain subnuclear particles during REM sleep might induce effects comparable to those elicited by available procedures of REM sleep deprivation. They might include repeated attempts to re-enter REM sleep, memory loss, and a higher net degree of synaptic potentiation. Comparable analyses of rats or mice raised in sensory and socially enriched environments could also be of interest.

## Conclusion

A detailed comparison of the current hypotheses on sleep memory processing proposed by SH and SHY has outlined their similarities and differences with regard to postulates, experimental approaches, data, and interpretations. According to SH the role of sleep cannot be reduced to a renormalization of experience-modified synapses, notwithstanding the value of the homeostatic process emphasized by SHY. Indeed, the primary function of sleep consists in sorting and retaining select memories, weakening or eliminating other memory traces, and integrating retained memories with preexisting memories. SHY may also be criticized for a number of other issues, including its failure to solve the implicit dilemma that emerges from framing synaptic renormalization in the current memory storage mechanism exclusively rooted in synaptic modifications. Indeed, if synapses are partly renormalized, modified synapses would accumulate in brain and hinder learning capacity. Conversely, if all synapses are renormalized, acquired memories would be deleted. The dilemma might be solved by assuming that memories are transferred to quantum subnuclear brain particles during REM. The hypothesis may be subjected to experimental verification.

## Conflict of interest statement

The author declares that the research was conducted in the absence of any commercial or financial relationships that could be construed as a potential conflict of interest.

## References

[B1] Abel T. Havekes R. Saletin J. M. Walker M. P. (2013). Sleep, plasticity and memory from molecules to whole-brain networks. Curr. Biol. 23, R774–R788. 10.1016/j.cub.2013.07.025 24028961 PMC4263505

[B2] Adams K. Oswald I. (1977). Sleep is for tissue restoration. J. R. Coll. Physicians Lond. 11, 376–388. 328867 PMC5368747

[B3] Allison T. Cicchetti D. V. (1976). Sleep in animals: ecological and constitutional correlates. Science 194, 732–734. 10.1126/science.982039 982039

[B4] Ambrosini M. V. Gambelunghe C. Mariucci G. Bruschelli G. Adami M. Giuditta A. (1994). Sleep-Wake variables and EEG power spectra in Mongolian gerbils and Wistar rats. Physiol. Behav. 56, 963–968. 10.1016/0031-9384(94)90330-1 7824598

[B5] Ambrosini M. V. Giuditta A. (2001). Learning and sleep: the sequential hypothesis. Sleep Med. Rev. 5, 477–490. 10.1053/smrv.2001.0180 12531155

[B6] Ambrosini M. V. Langella M. Gironi Carnevale U. A. Giuditta A. (1992). The sequential hypothesis on sleep function. III. The structure of post-acquisition sleep in learning and non learning rats. Physiol. Behav. 51, 217–226. 10.1016/0031-9384(92)90134-n 1557433

[B7] Ambrosini M. V. Mariucci G. Bruschelli G. Colarieti L. Giuditta A. (1995). Sequential hypothesis of sleep function. V. Lengthening of post-trial SS episodes in reminiscent rats. Physiol. Behav. 58, 1043–1049. 10.1016/0031-9384(95)00143-7 8577875

[B8] Ambrosini M. V. Mariucci G. Colarieti L. Bruschelli G. Carobi C. Giuditta A. (1993). The structure of sleep is related to the learning ability of rats. Eur. J. Neurosci. 5, 269–275. 10.1111/j.1460-9568.1993.tb00493.x 8261107

[B9] Ambrosini M. V. Sadile A. G. Gironi Carnevale U. A. Mattiaccio M. Giuditta A. (1988). The sequential hypothesis on sleep function. II. A correlative study between sleep variables and newly synthesized brain DNA. Physiol. Behav. 43, 339–350. 10.1016/0031-9384(88)90197-7 3174846

[B10] Anderson A. C. (1940). Evidences of reminiscence in the rat in maze learning. J. Comp. Physiol. Psychol. 30, 399–412 10.1037/h0055284

[B11] Ashapkin V. V. Romanov G. A. Tushmalova N. A. Vanyushin B. F. (1983). Selective DNA synthesis in the rat brain induced by learning. Biokhimija 48, 355–362.

[B12] Barry G. (2013). Lamarckian evolution explains human brain evolution and psychiatric disorders. Front. Neurosci. 7:224. 10.3389/fnins.2013.00224 24324395 PMC3840504

[B13] Crick F. (1984). Memory and molecular turnover. Nature 312:101. 10.1038/312101a0 6504122

[B14] Crick F. Mitchison G. (1983). The function of dream sleep. Nature 304, 111–114. 10.1038/304111a0 6866101

[B15] Crick F. Mitchison G. (1995). The function of dream sleep. Behav. Brain Res. 69, 147–155. 10.1016/0166-4328(95)00006-f 7546306

[B16] Dawlaty J. M. Ishizaki A. De A. K. Fleming G. R. (2012). Microscopic quantum coherence in a photosynthetic-light-harvesting antenna. Philos. Trans. A Math. Phys. Eng. Sci. 370, 3672–3691. 10.1098/rsta.2011.0207 22753820

[B17] Diekelmann S. Born J. (2010). The memory function of sleep. Nat. Rev. Neurosci. 11, 114–126. 10.1038/nrn2762 20046194

[B18] Eyman M. Cefaliello C. Mandile P. Piscopo S. Crispino M. Giuditta A. (2013). Training old rats selectively modulates synaptosomal protein synthesis. J. Neurosci. Res. 91, 20–29. 10.1002/jnr.23133 23086702

[B19] Ferrara E. Cefaliello C. Eyman M. De Stefano R. Giuditta A. Crispino M. (2009). Synaptic mRNAs are modulated by learning. J. Neurosci. Res. 87, 1960–1968. 10.1002/jnr.22037 19235900

[B20] Ficca G. Lombardo P. Rossi L. Salzarulo P. (2000). Morning recall of verbal material depends on prior sleep organization. Behav. Brain Res. 112, 159–163. 10.1016/s0166-4328(00)00177-7 10862947

[B21] Fishbein W. Gutwein B. M. (1977). Paradoxical sleep and memory storage processes. Behav. Biol. 19, 425–464. 10.1016/s0091-6773(77)91903-4 16586

[B22] Frank M. (2012). Erasing synapses in sleep: is it time to be SHY. Neural Plast. 2012:264378. 10.1155/2012/264378 22530156 PMC3317003

[B23] Frank M. G. (2013). Why I am not SHY: a reply to Tononi and Cirelli. Neural Plast. 2013:394946. 10.1155/2013/394946 23476811 PMC3583075

[B24] Frank M. G. (2014). Sleep and synaptic plasticity in the developing and adult brain. Curr. Top. Behav. Neurosci. [Epub ahead of print]. 10.1007/7854_2014_305 24671703 PMC7485264

[B25] Gais S. Plihal W. Wagner U. Born J. (2000). Early sleep triggers memory for early visual discrimination skills. Nat. Neurosci. 3, 1335–1339. 10.1038/81881 11100156

[B26] Giuditta A. (1977). “The biochemistry of sleep,” in Biochemical Correlates of Sleep Structure and Function, ed Davidson A. N. (New York: Academic Press), 293–337.

[B27] Giuditta A. (1985). “A sequential hypothesis for the function of sleep,” in Sleep ′84, eds Koella W. P. Ruther E. Schulz H. (Stuttgart: Fischer Verlag), 222–224.

[B28] Giuditta A. (2012). The nature of mind and its phylogenetic origin. Hum. Evol. 27, 281–290.

[B29] Giuditta A. Ambrosini M. V. Montagnese P. Mandile P. Cotugno M. Grassi Zucconi G.. (1995). The sequential hypothesis of the function of sleep. Behav. Brain Res. 69, 157–166. 10.1016/0166-4328(95)00012-I 7546307

[B30] Giuditta A. Ambrosini M. V. Scaroni R. Chiurulla C. Sadile A. (1985). Effect of sleep on cerebral DNA synthesized during shuttle-box avoidance training. Physiol. Behav. 34, 769–778. 10.1016/0031-9384(85)90376-2 4034717

[B31] Giuditta A. Chun J. T. Eyman M. Cefaliello C. Bruno A. P. Crispino M. (2008). Local gene expression in axons and nerve endings: the glia-neuron unit. Physiol. Rev. 88, 515–555. 10.1152/physrev.00051.2006 18391172

[B32] Giuditta A. Mandile P. Montagnese P. Piscopo S. Vescia S. (2003). “The role of sleep in memory processing: the sequential hypothesis,” in Sleep and Brain Plasticity, eds Maquet P. Smith C. Stickgold R. (Oxford: University Press), 157–178.

[B33] Giuditta A. Perrone Capano C. D’Onofrio G. Toniatti C. Menna T. Hydèn H. (1986). Synthesis of rat brain DNA during acquisition of an appetitive task. Pharmacol. Biochem. Behav. 25, 651–658. 10.1016/0091-3057(86)90155-3 3774832

[B34] Giuditta A. Rutigliano B. Vitale-Neugebauer A. (1980). Influence of synchronized sleep on the biosynthesis of RNA in two nuclear classes isolated from rabbit cerebral cortex. J. Neurochem. 35, 1259–1266. 10.1111/j.1471-4159.1980.tb08996.x 6160204

[B69] Grassi Zucconi G. Belia S. Menichini E. Castigli E. Giuditta A. (1986). Paradoxical sleep deprivation of the mother enhances DNA synthesis in fetal rat brain: autoradiographic and biochemical evidence. Int. J. Dev. Neurosci. 4, 169–178. 10.1016/0736-5748(86)90042-0 3455582

[B35] Hamenoff S. Penrose R. (2014). Consciousness in the universe: a review of the ‘Orch OR’ theory. Phys. Life Rev. 11, 39–78. 10.1016/j.plrev.2013.08.002 24070914

[B36] Horne J. A. (1977). Factors relating to energy conservation during sleep in mammals. Physiol. Psychol. 5, 403–408 10.3758/bf03337844

[B37] Horne J. A. (1981). The effects of exercise on sleep. Biol. Psychol. 12, 241–290. 10.1016/0301-0511(81)90001-6 7041996

[B38] Horne J. A. Walmsley B. (1976). Daytime visual load and the effects upon human sleep. Psychophysiology 13, 115–120. 10.1111/j.1469-8986.1976.tb00084.x 176675

[B39] Huppert F. A. Deutsch J. A. (1969). Improvement of memory with time. Quart. J. Exp. Psychol. 21, 267–271 10.1080/14640746908400221

[B40] Jaffard R. Destrade C. Soumiret-Mourat B. Carlo B. (1974). Time-dependent improvement of performance of appetitive tasks in mice. Behav. Biol. 11, 89–100. 10.1016/s0091-6773(74)90223-5 4833228

[B41] Langella M. Colarieti L. Ambrosini M. V. Giuditta A. (1992). The sequential hypothesis of sleep function. IV. A correlative analysis of sleep variables in learning and non learning rats. Physiol. Behav. 51, 227–238. 10.1016/0031-9384(92)90135-o 1557434

[B42] LePort A. K. Mattfeld A. T. Dickinson-Anson H. Fallon J. H. Stark C. E. Kruggel F.. (2012). Behavioral and neuroanatomical investigation of Highly Superior Autobiographical Memory (HSAM). Neurobiol. Learn. Mem. 98, 78–92. 10.1016/j.nlm.2012.05.002 22652113 PMC3764458

[B43] Mackiewicz M. Shockley K. R. Romer M. A. Galante R. J. Zimmerman J. E. Naidoo N.. (2007). Macromolecule biosynthesis: a key function of sleep. Physiol. Genomics 31, 441–457. 10.1152/physiolgenomics.00275.2006 17698924

[B44] Mandile P. Giuditta A. Romano F. Montagnese P. Piscopo S. Cotugno M.. (2003). Waking EEG power spectra in the rat: correlations with training performance. Brain Res. Cogn. Brain Res. 17, 94–105. 10.1016/s0926-6410(03)00084-3 12763196

[B45] Mandile P. Vescia S. Montagnese P. Piscopo S. Cotugno M. Giuditta A. (2000). Post-trial sleep sequences including transition sleep are involved in avoidance learning of adult rats. Behav. Brain Res. 112, 23–31. 10.1016/s0166-4328(00)00158-3 10862932

[B46] Mariucci G. Bruschelli G. Colarieti L. Gambelunghe C. Ambrosini M. V. (1998). Wistar rats retain for months memory of active avoidance learned behavior. Ital. J. Zool. 65, 311–314 10.1080/11250008809386764

[B47] Pearlman C. A. (1979). REM sleep and information processing: evidence from animal studies. Neurosci. Biobehav. Rev. 3, 57–68 10.1016/0149-7634(79)90034-4

[B48] Pelc S. R. (1972). Metabolic DNA in ciliated protozoa, salivary gland chromosomes and mammalian cells. Int. Rev. Cytol. 32, 327–355. 10.1016/s0074-7696(08)60344-7 4623842

[B49] Pelc S. R. Viola-Magni M. P. (1969). III. Decrease of labeled DNA in cells of the adrenal medulla after intermittent exposure to cold. J. Cell Biol. 42, 460–468. 10.1083/jcb.42.2.460 5792334 PMC2107685

[B50] Perrone Capano C. D’Onofrio G. Giuditta A. (1982). DNA turnover in rat cerebral cortex. J. Neurochem. 38, 52–56. 10.1111/j.1471-4159.1982.tb10852.x 7108534

[B51] Pigarev I. N. (1994). Neurons of visual cortex respond to visceral stimulation during slow wave sleep. Neuroscience 62, 1237–1243. 10.1016/0306-4522(94)90355-7 7845596

[B52] Pigarev I. N. Bagaev V. A. Levichkina E. V. Fedorov G. O. Busigina I. I. (2013). Cortical visual areas process intestinal information during slow-wave sleep. Neurogastroenterol. Motil. 25, 268–275. 10.1111/nmo.12052 23216826

[B53] Pigarev I. N. Pigarev M. L. (2014). Partial sleep in the context of augmentation of brain function. Front. Syst. Neurosci. 8:75. 10.3389/fnsys.2014.00075 24822040 PMC4013465

[B54] Piscopo S. Mandile P. Montagnese P. Cotugno M. Giuditta A. Vescia S. (2001). Trains of sleep sequences are indices of learning capacity in rats. Behav. Brain Res. 120, 13–21. 10.1016/s0166-4328(00)00360-0 11173081

[B55] Reich P. Geyer S. J. Karnovsky M. L. (1972). Metabolism of brain during sleep and wakefulness. J. Neurochem. 19, 487–497. 10.1111/j.1471-4159.1972.tb01358.x 4334501

[B56] Reinis S. Lamble R. W. (1972). Labeling of brain DNA by ^3^H-thymidine during learning. Physiol. Chem. Phys. 4, 335–338. 4680783

[B57] Ribeiro S. (2012). Sleep and plasticity. Pflugers Arch. 463, 111–120. 10.1007/s00424-011-1031-5 21947578 PMC3256318

[B58] Roffwarg H. P. Muzio J. N. Dement W. C. (1966). Ontogenetic development of the human sleep-dream cycle. Science 152, 604–619. 10.1126/science.152.3722.604 17779492

[B59] Rudenko A. Tsai L.-H. (2014). Epigenetic regulation in memory and cognitive disorders. Neuroscience 264, 51–63. 10.1016/j.neuroscience.2012.12.034 23291453

[B60] Shapiro C. M. Bortz R. Mitchell D. Bartel P. Jooste P. (1981). Slow-wave sleep: a recovery period after exercise. Science 214, 1253–1254. 10.1126/science.7302594 7302594

[B61] Stickgold R. James L. Hobson J. A. (2000). Visual discrimination learning requires sleep after training. Nat. Neurosci. 3, 1237–1238. 10.1038/81756 11100141

[B62] Timofeev I. (2011). Neuronal plasticity and thalamocortical sleep and waking oscillations. Prog. Brain Res. 193, 121–144. 10.1016/b978-0-444-53839-0.00009-0 21854960 PMC3250382

[B63] Tononi G. Cirelli C. (2003). Sleep and synaptic homeostasis: a hypothesis. Brain Res. Bull. 62, 143–150. 10.1016/j.brainresbull.2003.09.004 14638388

[B64] Tononi G. Cirelli C. (2006). Sleep function and synaptic homeostasis. Sleep Med. Rev. 10, 49–62. 10.1016/j.smrv.2005.05.002 16376591

[B65] Tononi G. Cirelli C. (2014). Sleep and the price of plasticity: from synaptic and cellular homeostasis to memory consolidation and integration. Neuron 81, 12–34. 10.1016/j.neuron.2013.12.025 24411729 PMC3921176

[B66] Vescia S. Mandile P. Montagnese P. Romano F. Cataldo G. Cotugno M.. (1996). Baseline transition sleep and associated sleep episodes are related to the learning ability of rats. Physiol. Behav. 60, 1513–1525. 10.1016/s0031-9384(96)00302-2 8946500

[B67] Vitale-Neugebauer A. Giuditta A. Vitale B. Giaquinto S. (1970). Pattern of RNA synthesis in rabbit cortex during sleep. J. Neurochem. 17, 1263–1273. 10.1111/j.1471-4159.1970.tb03375.x 4318653

[B68] Walker J. M. Berger R. J. (1980). Sleep as an adaptation for energy conservation functionally related to hibernation and shallow torpor. Prog. Brain Res. 53, 255–278. 10.1016/s0079-6123(08)60068-0 7005945

